# Stewarding Recovery from the Opioid Crisis Through Health System Initiatives

**DOI:** 10.5811/westjem.2018.11.39013

**Published:** 2019-02-02

**Authors:** Jeanmarie Perrone, Scott G. Weiner, Lewis S. Nelson

**Affiliations:** * Perelman School of Medicine at the University of Pennsylvania, Department of Emergency Medicine, Philadelphia, Pennsylvania; † Brigham and Women’s Hospital, Department of Emergency Medicine, Boston, Massachusetts; ‡ Rutgers New Jersey Medical School, Department of Emergency Medicine, Newark, New Jersey

## Abstract

As the consequences of liberal opioid prescribing have become apparent, efforts to address the role of the health care system in supporting more balanced opioid use and the prevention and treatment of opioid use disorder have increased. Developing a unified and multidisciplinary approach can lead to an integrated care model that emphasizes primary prevention, harm reduction, and transition to life-sustaining treatment while also maintaining attentiveness to effective pain management. A model for this, which follows the nomenclature in proscribing antimicrobial use, is the development of an opioid stewardship program. Such programs allow for the integration of diverse perspectives and new mandates and uses a patient-centered approach with an iterative evaluation process. We describe a group of adoptable efforts that have been utilized successfully at our institutions and may be adapted and optimized to the needs and resources of other hospitals and health care systems.

The tragic opioid epidemic in the United States (U.S.) claimed 115 lives a day in 2016.^1^ Multiple factors have contributed to the escalating opioid death toll, particularly a rapid and substantial increase in fentanyl-related deaths.^2^ Hidden in the well-publicized, escalating fentanyl fatality data is the fact that prescription opioid deaths also continue to rise, albeit more slowly.^3^ Furthermore, opioid prescribing, with its associated consequences of long-term opioid use including addiction, has fallen only modestly despite significant efforts.^4^ Mitigation of the prescription opioid epidemic will only be achieved when analgesic-prescribing pathways minimize opioid initiation, patients prescribed opioids are carefully monitored, and patients with existing opioid use disorder (OUD) are ushered into treatment. We believe that hospitals and health systems are essential components of the solution and describe a framework to create a comprehensive opioid stewardship program that can improve patient outcomes, quality of care, and regulatory compliance. Such a program aligns with the shifting societal attitudes and awareness of the risk and consequences of opioid addiction and the role of health systems in health promotion in their communities.

To date, some large health systems such as the Veterans Administration, have developed systematic approaches to pain management that balance the public and regulatory pressures to standardize opioid prescribing while addressing patient goals and safety.^5^ Recommendations from the Joint Commission that went into effect January 1, 2018, mandate that all healthcare facilities now implement leadership teams and performance improvement processes to address safe opioid prescribing.^6^ The National Quality Forum released guidelines to measure and respond to new changes in opioid management in March 2018.^7^ We highlight the initiatives implemented in our health systems to meet these new mandates. We recommend organizing and expanding these efforts into a formal opioid stewardship program (OSP), a term mirroring the infectious disease platforms promoting judicious antibiotic use. OSPs provide the necessary framework to identify gaps in quality and develop and implement a tripartite change of culture and practice: 1) encourage use of non-opioids as first-line treatment for pain; 2) provide pathways to safer opioid use when opioids are indicated; and 3) identify and engage patients with OUD into treatment. These are described in more detail below as well as in Table 1.

The three authors, who have collaborated extensively on the mitigation of opioid-related consequences, have gained valuable insights following implementation of OSPs at their academic institutions. Through shared experiences and an iterative process, each has developed a successful OSP that addresses the needs of their respective institutions. A successful OSP requires executive support and rigorous project management, oversight by key clinical leaders, and integration of multidisciplinary stakeholders as shown in Table 2. Although the program can be directed by a number of specialties, our experiences as emergency physicians show that we are well suited to the task because of our experience treating patients with acute and chronic pain, as well as OUD. Being hospital based, the emergency department (ED) is well integrated into the administrative structure and routinely interacts with the other clinical services.

The ability to use information technology (IT) resources was critical to provide benchmarking of opioid use, collect timely metrics, and build best practice, clinical decision support tools. Dissemination of new pathways and protocols across the institution was addressed by the authors through academic detailing (e.g., individual meetings, grand rounds) to departments and creation of an institutional OSP website (e.g., bcore.brighamandwomens.org).

## Limiting Opioid Initiation: Keep Opioid-naïve Patients Opioid Naïve When Possible

We individually developed pain management pathways and order sets that deemphasize opioid use using an iterative consensus process by engaged providers starting with specialties with high utilization (e.g., primary care, emergency medicine). For procedure-focused specialties such as orthopedics and general surgery, direct, procedure-specific modifications in pre- and post-procedure prescribing were similarly created. Patient feedback, both obtained during deliberate rounding and through direct post-procedure assessments at three to seven days suggested opportunities to “right size” the number of pills prescribed while still assuring the provision of adequate pain management. Certain states (e.g., Massachusetts. New York, New Jersey) have placed regulatory controls on initial opioid prescribing that dovetailed with the implementation of the OSP guidelines.

The recently modified pain questions in the Hospital Consumer Assessment of Healthcare Providers and Systems (HCAHPS) are an attempt to shift the focus from pain management outcomes, which are often medication-centered, toward adequacy of pain assessment.^8^ To support this, institution-specific multidisciplinary education modules emphasizing the role of opioid alternatives can be created, aligning with the U.S. Food and Drug Administration’s 2017 blueprint for the treatment of patients with pain.^9^ Programs should highlight the significant risk for developing long-term opioid use and the recognition that our ability to predict who may develop an OUD following even minimal (one-day) opioid exposure is limited.^10^ Electronic health record (EHR) decision support can prioritize non-opioid and non-pharmacologic pain management options and redirect providers who have been trained to practice using opioids as a first-line pain relief option.

## Using Opioids When, and Only When, an Opioid is Indicated

OSPs identified resources from local, state, and federal governmental agencies and professional organizations to guide appropriate and safe opioid use when indicated. Such guidance addressed various aspects of pain, such as in the post-operative setting or managing acute severe pain in the ED and were adopted or modified to be institution or procedure specific.^11^ Guidelines were implemented with corresponding outcome measurements to allow incremental standardization of opioid prescribing practices. Monitoring outcomes highlights success, such as a recent pilot in Colorado designed to reduce ED opioid prescribing by 15% through implementation of standardized alternative pain-management strategies that exceeded expectation (36% reduction).^12^ They similarly allow for assessment of adverse outcomes, as noted by an effort to use evidence-based, postoperative prescribing guidelines led to a 63% reduction in opioid prescribing,^13^ and lowering the EHR default reduced opioid prescribing by about one-third,^14^ both without an increase in requests for medication refills.

Attention to the frequent use of opioids for the treatment of chronic pain is of paramount importance given the increasingly recognized role of hyperalgesia in perpetuating continued use. In accordance with Centers for Disease Control and Prevention guidelines, health systems can facilitate compliance with opioid use agreements, urine drug monitoring for both compliance (e.g., diversion) and prohibited drug use, prevent benzodiazepine co-prescribing, and performance of functional outcome assessments. Safe-use education should become part of opioid-specific discharge instructions including emphasis on appropriate storage and disposal of remaining medication. For those patients already managed on high-dose opioids for their chronic pain, we encouraged the creation of pathways for dose reduction to the recommended dose of 90 morphine milligram equivalents (MME).^11^ For patients unable or unwilling to undergo gradual dose tapering, they were cautiously maintained on their dose and the recommendations of existing pain-management guidelines for monitoring were followed.

OSPs can leverage EHRs to develop dashboards of opioid-use patterns by department or prescriber with the goal of reducing variability as a marker of quality care. OSPs can provide oversight of regulatory changes and evolving state laws affecting prescribing, such as mandatory prescription drug monitoring program (PDMP) queries, consent for minors for opioid prescriptions, and prompts for the initiation of controlled medication agreements. Providing decision support, order sets, prescribing defaults, maximum MMEs, and using nudges, reminders, and best practice alerts are efforts that helped reduce the initiation of opioids or limit the dose and duration provided.^15^

## Treating Patients with Opioid Use Disorder

OSPs must expand recognition and timely management of patients with OUD. Compassionate care of hospitalized patients suffering from complications of illicit opioid use (e.g., endocarditis, abscess) emphasizing opioid agonist therapy to mitigate opioid withdrawal, reduce premature self-discharge and readmission, enhance opportunities to transition to methadone or buprenorphine, and improve other medication adherence such as antibiotic therapy is essential.

Additionally, resources should be allocated for “warm handoffs” to addiction treatment programs using hospital-based substance use disorder clinics and peer recovery coaches to engage patients into treatment. A comprehensive approach to mitigating opioid harm includes naloxone prescribing and distribution programs for at-risk individuals. Primary care providers should be supported to integrate buprenorphine prescribing into their practices to expand capacity for referrals and allow patients to find evidence-based treatment within the health system home.^16^

These concepts broaden existing new mandates to address multiple, intertwined morbidities associated with opioid use. They implement best practices and necessary resources to guide health systems tasked with this challenging work. The severity of the crisis and the rapidly changing regulatory and public health landscape dictate that sensible change must start immediately. Although the mandate for action is national, a substantial component of the solution is local. Hospitals and health systems are uniquely poised to create an integrated care model that emphasizes primary prevention, harm reduction, and transition to life-sustaining treatment. OSPs provide a specific mechanism to integrate many perspectives and requirements into a process to reduce consequences of excessive and inappropriate opioid use, and assure that those in pain receive safe and effective care.

## Figures and Tables

**Table 1:** Roadmap to the implementation of an opioid stewardship program (OSP).

The leadership team: Multidisciplinary stakeholder input: representatives from primary care, anesthesiology, emergency medicine, psychiatry, surgery, and pharmacy with executive support from the chief medical officer, chief quality officer, and chief nursing officer. Potential task forces/subcommittees: Guidelines and pathways Education and outreach Legal and compliance Information technology The missions: Limit opioid initiation Rationalize expectations among patients for pain and pain relief Create prescribing guidelines Standardize order sets emphasizing non-opioid approaches as first and second line Education and best practice alerts about non-opioid and non-pharmacologic (multimodal) therapies Community intervention/education programs to discourage diversion and non-medical use Improve the safety of opioid use Leverage the electronic health record Best practice alerts for compliance with safe opioid treatment guidelines and state/federal regulations. Integrate prescription drug monitoring program access Track and nudge providers and departments using dashboards and e-alerts following compliance trends. Default formulations (immediate release), doses, and schedules for opioid orders and prescriptions Prompt at discharge to educate patients about safe storage, appropriate disposal and naloxone Create pain management strategies Standardize short-term dosing based on common diagnoses and procedures Compliance with state regulations and documentation requirements Create monitoring parameters for patients receiving high-dose opioids Develop systems or registries to check for presence of opioid use agreements, urine drug-screen results, maximum morphine equivalent dosing, and rates of co-prescribed benzodiazepines Create endpoints for acceptable opioid use (e.g., maximum of 90 morphine milligram equivalents/day) and exit strategies such as weaning Other activities Disseminate educational modules on pain assessment and opioid stewardship to meet Joint Commission recommendations Integrate clinical pharmacists into medication management Treating patients with opioid use disorder Operationalize addiction management Increase screening for opioid use disorder at admission and in primary care practices Reduce barriers for the use of buprenorphine or methadone to mitigate opioid withdrawal in hospitalized patients Organize resources to improve hand-offs to settings that provide opioid agonist therapy Implement harm reduction strategies Naloxone distribution or prescribing Certified recovery specialists/peer navigators and other social services Family and community engagement processes Safe practices (clean syringes, counsel about risk of infection)

**Table 2:** An example organizational structure for an academic health center opioid stewardship program.

Steering committee Chair or co-chairs Chair of anesthesiology (or designee) Chair of emergency medicine (or designee) Chair of internal medicine (or designee) Chair of psychiatry (or designee) Chair of surgery (or designee) Chief medical officer Chief nursing officer Chief information office Graduate medical education director/designated institutional official Pharmacy director Project manager Quality/safety Tasks Prioritize efforts Populate task forces Develop initial expectations and metrics Guide committee efforts with periodic meetings and oversight Evaluate metrics and suggest improvements
Guidelines and pathways/pain management Chair or co-chairs One representative from each: Ambulatory care/primary care Emergency medicine Hospice/palliative care Internal medicine/hospitalist Nursing Oncology Orthopedic surgery Pain medicine/anesthesia Pediatrics Pharmacy Rheumatology Surgery Tasks Assessment of current state Benchmarking of progress Guideline development for pain management Implementation
Addiction and harm reduction committee Chair or co-chairs One representative from each: Addiction psychiatry/addiction medicine Ambulatory care/primary care Emergency medicine Internal medicine/hospitalist Nursing Pain medicine/anesthesia Pharmacy Social work Surgery Tasks Benchmarking current status Capacity development Process improvement Implement harm reduction efforts
Quality and information technology Chair or co-chairs: Chief medical information officer Quality/safety leader Information technology Physician leader Nurse leader Pharmacy leader Other committee chairs Tasks Define the scope of the problem Develop and implement recommendation with other committees Analyze capacity for addiction treatment Process improvement for addiction management Assess rates of hospitalized patients with opioid use disorder who leave against medical advice as these are missed opportunities to improve withdrawal care Provide strategies for opioid withdrawal management with buprenorphine and methadone
Education and outreach Chair or co-chairs Physician leader Nursing leader Pharmacy leader Graduate medical education representative Tasks Implement an awareness campaign Implement a continuing education program Collect feedback from constituencies
